# Fatality rate, risk factors, and functional decline in peritoneal dialysis patients with coronavirus disease 2019: A nationwide cohort study

**DOI:** 10.3389/fmed.2022.1051448

**Published:** 2022-11-17

**Authors:** Piyatida Chuengsaman, Sarinya Boongird, Phongsak Dandecha, Thiravat Hemachudha, Tanawin Nopsopon, Talerngsak Kanjanabuch, Suchai Sritippayawan, Surasak Kantachuvesiri

**Affiliations:** ^1^Banphaeo-Charoenkrung Peritoneal Dialysis Center, Banphaeo Dialysis Group, Banphaeo Hospital, Bangkok, Thailand; ^2^Division of Nephrology, Department of Medicine, Faculty of Medicine Ramathibodi Hospital, Mahidol University, Bangkok, Thailand; ^3^Division of Nephrology, Department of Internal Medicine, Prince of Songkla University, Hat Yai, Thailand; ^4^World Health Organization Collaborating Centre for Research and Training on Viral Zoonoses, King Chulalongkorn Memorial Hospital, Faculty of Medicine, Chulalongkorn University, Bangkok, Thailand; ^5^Department of Preventive and Social Medicine, Faculty of Medicine, Chulalongkorn University, Bangkok, Thailand; ^6^Peritoneal Dialysis Excellence Center, Chulalongkorn University, Bangkok, Thailand; ^7^Center of Excellence in Kidney Metabolic Disorders, Faculty of Medicine, Chulalongkorn University, Bangkok, Thailand; ^8^Division of Nephrology, Department of Medicine, Faculty of Medicine, Chulalongkorn University, Bangkok, Thailand; ^9^Division of Nephrology, Department of Medicine, Faculty of Medicine Siriraj Hospital, Mahidol University, Bangkok, Thailand

**Keywords:** coronavirus disease 2019 (COVID-19), death, dialysis, functional status, mortality, outcome, peritoneal dialysis, severe acute respiratory syndrome coronavirus 2 (SARS-CoV-2)

## Abstract

**Background:**

The fatality rates and factors associated with death from coronavirus disease 2019 (COVID-19) in hemodialysis patients have been extensively investigated. However, data on peritoneal dialysis (PD) patients remain scarce.

**Materials and methods:**

In this nationwide cohort study, we assessed the 28-day COVID-19-related fatality rate in PD patients between August 2021 and July 2022 using data from the InCov19-PD registry. Predictors associated with death were evaluated using a multivariable Cox regression model. Changes in functional status before and during COVID-19 were also examined.

**Results:**

A total of 1,487 eligible participants were evaluated. During the study period, 196 participants died within 28 days after COVID-19 diagnosis (case fatality rate: 13%). In a multivariable Cox regression model, an increased risk of death within 28 days after COVID-19 diagnosis among PD patients was independently associated with functional impairment during COVID-19 [adjusted hazard ratio (HR) 2.46, 95% confidence interval (CI) 1.59–3.81], SARS-CoV-2 infection with the Delta variant (HR 2.23, 95% CI 1.55–3.21), and the need for respiratory support (HR 7.13, 95% CI 3.74–13.57) (*p* < 0.01 for all). Conversely, the number of COVID-19 vaccines administered (HR 0.69, 95% CI 0.55–0.87; *p* = 0.001) and receiving corticosteroid therapy during COVID-19 (HR 0.72, 95% CI 0.54–0.97; *p* = 0.03) were associated with a decreased risk of death within 28 days after COVID-19 diagnosis. The number of functionally independent PD patients dropped from 94% at baseline to 63% during COVID-19 (*p* < 0.01).

**Conclusions:**

The COVID-19-related 28-day fatality rate was high among PD patients. The predictors of COVID-19-related death in PD patients were similar to those in hemodialysis patients. During COVID-19, PD patients commonly experienced functional deterioration.

## Introduction

The coronavirus disease 2019 (COVID-19) pandemic posed unprecedented challenges for patients with end-stage kidney disease (ESKD) requiring kidney replacement therapy (KRT). Numerous studies have demonstrated that the case fatality rates of COVID-19 in ESKD patients are significantly higher than in the general population (1–4). To mitigate the risk of severe acute respiratory syndrome coronavirus 2 (SARS-CoV-2) infection in ESKD patients, several interventions have been implemented, including personal protective behaviors, administration of COVID-19 vaccines, and home containment measures. Some nephrologists consider peritoneal dialysis (PD) as an appealing modality of KRT during the COVID-19 pandemic given the lower risk of exposure to other patients and dialysis personnel in hemodialysis (HD) units (4–7). However, data on the COVID-19-related fatality rate and its impact on clinical outcomes in PD patients remain poorly understood.

Among ESKD patients diagnosed with COVID-19, those on chronic PD therapy have received much less attention than those on the other dialysis modalities. The majority of the COVID-19 mortality data and clinical outcomes are derived from the HD and kidney transplant (KT) populations (2, 8, 9). A few studies, primarily case series or small cohorts with a limited number of PD patients, have examined those outcomes of the PD population (10–13). Earlier studies showed that fatigue and myalgia were prevalent among ESKD patients diagnosed with COVID-19, ranging from 3 to 63% (14). Those symptoms may impair the patient’s functional status and ability to continue PD treatment. Consequently, it is necessary to understand the impacts of COVID-19 on mortality, clinical outcomes, changes in functional status, and alterations in basic PD treatment care among PD patients diagnosed with COVID-19. Such information can inform healthcare professionals, caregivers, and patients on modifying PD-related treatment plans, vaccine strategies, and interventions for this vulnerable group.

Herein, we sought to determine the 28-day case fatality rate and predictors of COVID-19-related death among patients on chronic PD therapy. Alterations in patients’ functional status and pattern of routine PD care, focusing on PD bag exchange, were also investigated.

## Materials and methods

### Study design and population

This is a prospective observational nationwide cohort using data from the InCov19-PD registry, a national surveillance registry that assessed the clinical outcomes and health impacts of SARS-CoV-2 infection in Thai PD patients. Under the auspices of the Nephrology Society of Thailand (NST), the InCov19-PD registry prospectively collected data from August 2021 to July 2022 on PD patients diagnosed with COVID-19. Patients were included in the study if they were PD patients who tested positive for SARS-CoV-2, were at least 12 years old, and had received chronic PD treatment for at least 1 month before COVID-19 diagnosis. For PD patients who had multiple COVID-19 episodes, only the initial COVID-19 episodes were included in the study. Any PD patients who had no documented clinical outcomes at day 28 after COVID-19 diagnosis, aged less than 12 years old, or commencing PD treatment for acute kidney injury were excluded from the study. A COVID-19 diagnosis was confirmed either by a positive SARS-CoV-2 real-time reverse transcription polymerase chain reaction test or a rapid antigen test kit (ATK) on samples obtained from the nasopharyngeal swab.

### Ethics approval

The Institutional Review Board of the Faculty of Medicine, Chulalongkorn University, Bangkok, Thailand approved this study (IRB No. 0298/65). All participants provided written informed consent before enrollment. The study was conducted following the principles laid out in the declaration of Helsinki.

### Outcomes and definitions

The primary outcome was the 28-day case fatality rate of PD patients with confirmed COVID-19 in the InCov19-PD registry. The COVID-19 confirmation date was utilized as the index date when calculating the 28-day case fatality rate. Survivors were participants who were still alive 28 days following the COVID-19 confirmation date. The remaining individuals who died within 28 days as a result of COVID-19 were classified as non-survivors.

Secondary objectives included identifying predictors of COVID-19-related death within 28 days following a confirmed COVID-19 diagnosis. The impacts of COVID-19 on hospitalization rate, the need for respiratory support, and changes in patient functional status and patterns of PD bag exchange during COVID-19 were also examined. The baseline functional status of patients before and at the time of COVID-19 diagnosis was evaluated and categorized as independent, partially dependent, or totally dependent on a caregiver.

### Data collection

At the end of July 2021, physicians and PD nurses from all NST-registered PD facilities were invited to voluntarily participate in this study. The InCov19-PD registry collected patient-level and facility-level data from all voluntarily participating facilities and study participants using a standard process and standardized data collection instruments. The NST verified the index case by contacting the treating physicians and reference PD nurses. Responsible physicians or PD nurses provided the NST with demographic data, comorbidities, details of PD prescriptions, COVID-19 vaccination status and regimens, laboratory parameters at the time of COVID-19 diagnosis, identified SARS-CoV-2 strains, initial and subsequent treatments, and COVID-19-related complications *via* an online database system. Clinical outcomes were evaluated at baseline and monitored until the patient died or until day 28 after COVID-19 diagnosis, whichever came first. The baseline functional status of PD patients and assistance with PD bag exchange before and during COVID-19 were obtained from the index patient and/or their family members using a semi-structured questionnaire. The COVID-19 confirmation date was utilized as the index date for each participant’s baseline characteristics, laboratory data, functional status, and vaccination record. All participants were followed until death, recovery, or hospital discharge.

### Statistical analysis

The categorical variables were described as the frequency with percentage, while the continuous variables were presented as mean with standard deviation (SD). Baseline demographic data and laboratory parameters between the survivor and non-survivor groups were compared using the Chi-square test and Fisher’s exact test for categorical factors, and the independent *t*-test for continuous variables. The 28-day case fatality rate of the overall cohort was computed. Fatality outcome was analyzed by survival analyses using Kaplan–Meier curves with a Log-rank test. All included patients had a complete follow-up, thus there was no censoring for missing outcome data. The associations between the covariates and the 28-day case fatality outcome were first evaluated using univariable Cox proportional hazard regression and subsequently adjusted for age. The multivariable Cox regression model was conducted using backward elimination and list-wise exclusion of missing data. All variables with age-adjusted *p*-values of less than 0.10 were candidates for the multivariable model. The least significant variables were removed repeatedly from the model until all variables had *p*-values below 0.05. The proportional hazards assumption was verified using Schoenfeld residuals and plots. Data were analyzed using R 4.0.5 (R Core Team, Vienna). *P*-values less than 0.05 were statistically significant.

## Results

### Baseline clinical characteristics

**FIGURE 1:**
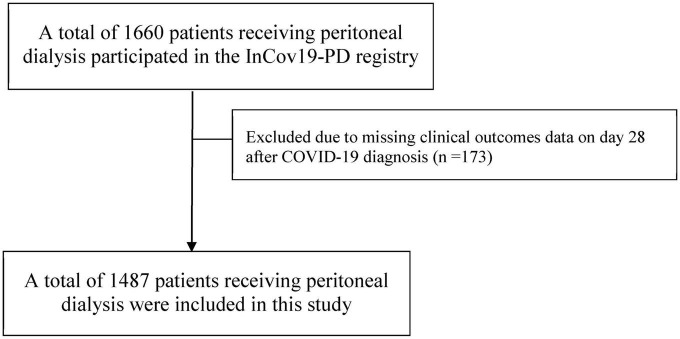
Study flow diagram indicating the study population and cohort enrollment process.

The InCov19-PD registry documented a total of 1,660 PD patients diagnosed with COVID-19 from August 2021 to July 2022. Of those, 1,487 eligible participants with a complete clinical record of outcomes on day 28 after COVID-19 diagnosis and meeting other inclusion criteria were included in the analysis (Figure 1). Baseline characteristics, laboratory parameters, PD vintage and prescriptions, and functional and vaccination status of study participants in the survivor and non-survivor groups are presented in Table 1. The overall mean (SD) age of study participants and dialysis vintage (SD) was 55.8 (14.4) years and 2.6 (2.4) years, respectively. At baseline, only 60% of PD patients in this cohort had received at least one dose of the COVID-19 vaccine. The median (interquartile range, IQR) follow-up time from the COVID-19 diagnosis to death in the non-survivor group was 9 (5–14) days.

**TABLE 1:** Characteristics of participants at baseline and during COVID-19 by mortality status on day 28 after COVID-19 diagnosis.

Baseline demographic data *n* (%) or mean (SD)	Total (*n* = 1487)	Non-survivor (*n* = 196)	Survivor (*n* = 1291)	*p*-value
Age, years	55.8 ± 14.4	61.5 ± 12.6	54.8 ± 14.4	<0.001
BMI, kg/m^2^	24.0 ± 4.4	24.4 ± 4.8	23.9 ± 4.4	0.14
Comorbidities, *n* (%)				
Diabetes	715 (55%)	135 (69%)	580 (52%)	< 0.001
Hypertension	1152 (88%)	174 (89%)	978 (88%)	0.89
Dyslipidemia	366 (28%)	69 (35%)	297 (27%)	0.02
Others	140 (11%)	35 (19%)	105 (10%)	0.08
Urine output × 100 ml/day	3.7 ± 4.2	3.5 ± 3.8	3.7 ± 4.2	0.49
Anuria, *n* (%)	510 (39%)	74 (38%)	436 (39%)	0.66
Automated PD, *n* (%)	78 (6%)	12 (6%)	66 (6%)	0.93
PD vintage, years	2.6 ± 2.4	2.5 ± 2.3	2.6 ± 2.4	0.36
PD volume × 1,000 ml/day	8.2 ± 1.1	8.3 ± 1.2	8.2 ± 1.1	0.18
Assistance with PD bag exchange, *n* (%)				< 0.001
None	758 (59%)	79 (41%)	679 (62%)	
Partially	465 (36%)	95 (50%)	370 (34%)	
Completely	65 (5%)	17 (9%)	48 (4%)	
Functional status, *n* (%)				< 0.001
Independent	1224 (94%)	163 (83%)	1061 (96%)	
Partially dependent	40 (3%)	22 (11%)	18 (2%)	
Totally dependent	38 (3%)	11 (6%)	27 (2%)	
Hemoglobin, g/dL	10.0 ± 2.0	9.9 ± 2.0	10.0 ± 2.0	0.35
Serum albumin, g/dL	3.2 ± 0.6	3.0 ± 0.7	3.2 ± 0.6	< 0.001
Number and regimen of COVID-19 vaccines administered^a^, *n* (%)	1.2 ± 1.0	0.4 ± 0.8	1.3 ± 1.0	< 0.001
None	529 (42%)	149 (76%)	380 (35%)	< 0.001
Single dose	133 (10%)	26 (13%)	107 (10%)	
Two doses	513 (40%)	17 (9%)	496 (46%)	
Two doses of the inactivated SARS-CoV-2 vaccine	55	3	52	
Two doses of the viral vector-based vaccine	161	4	157	
Two doses of the mRNA- based vaccine	62	1	61	
Any heterologous vaccination combination	235	9	226	
Three or more doses of any vaccination combination	93 (7%)	4 (2%)	89 (8%)	
Unknown	34 (3%)	0 (0%)	34 (3%)	
Participants who received at least one dose of the mRNA-based COVID-19 vaccine, *n* (%)	114 (9%)	5 (3%)	109 (10%)	0.001
**During COVID-19**				
Median follow-up time [IQR], days	28 [28–28]	9 [5–14]	28 [28–28]	
Hospitalization, *n* (%)	921 (62%)	191 (97%)	730 (57%)	< 0.001
COVID-19 variants^b^, *n* (%)				< 0.001
Delta	373 (30%)	137 (73%)	236 (22%)	
Omicron	872 (70%)	51 (27%)	821 (78%)	
**Treatment, *n* (%)**				
Oxygen support	536 (41%)	183 (93%)	353 (32%)	< 0.001
High flow nasal cannula or intubation	163 (13%)	118 (60%)	45 (4%)	< 0.001
Favipiravir	1156 (89%)	170 (87%)	986 (89%)	0.32
Lopinavir/Ritonavir	35 (3%)	12 (6%)	23 (2%)	0.001
Remdesivir	54 (4%)	16 (8%)	38 (3%)	0.002
Corticosteroid	334 (26%)	89 (45%)	245 (22%)	< 0.001
Andrographolide	92 (7%)	13 (7%)	79 (7%)	0.80
Automated PD, *n* (%)	137 (11%)	39 (20%)	98 (9%)	< 0.001
Assistance with PD bag exchange^a,c^, *n* (%)				< 0.001
None	652 (50%)	28 (14%)	624 (56%)	
Caregiver	398 (31%)	52 (27%)	346 (31%)	
Nurse	252 (19%)	116 (59%)	136 (12%)	
Functional status^a,c^, *n* (%)				< 0.001
Independent	814 (63%)	47 (24%)	767 (69%)	
Partially dependent	401 (31%)	103 (53%)	298 (27%)	
Totally dependent	87 (7%)	46 (23%)	41 (4%)	

Values are mean (SD) unless otherwise indicated. Patients’ characteristics between the non-survivor and survivor groups were compared using the independent *t*-test and Fisher’s exact test for continuous and categorical variables, respectively. BMI was calculated from weight in kilograms divided by height squared. BMI, body mass index; COVID-19, coronavirus disease 2019; IQR, interquartile range; mRNA, messenger ribonucleic acid; PD, peritoneal dialysis; SARS-CoV-2, severe acute syndrome coronavirus 2; SD, standard deviation.

^a^Total percentage is not equal to 100% due to rounding.

^b^Evaluated in 1,245 participants.

^C^Evaluated in 1,302 participants.

### The 28-day case fatality rate

**FIGURE 2:**
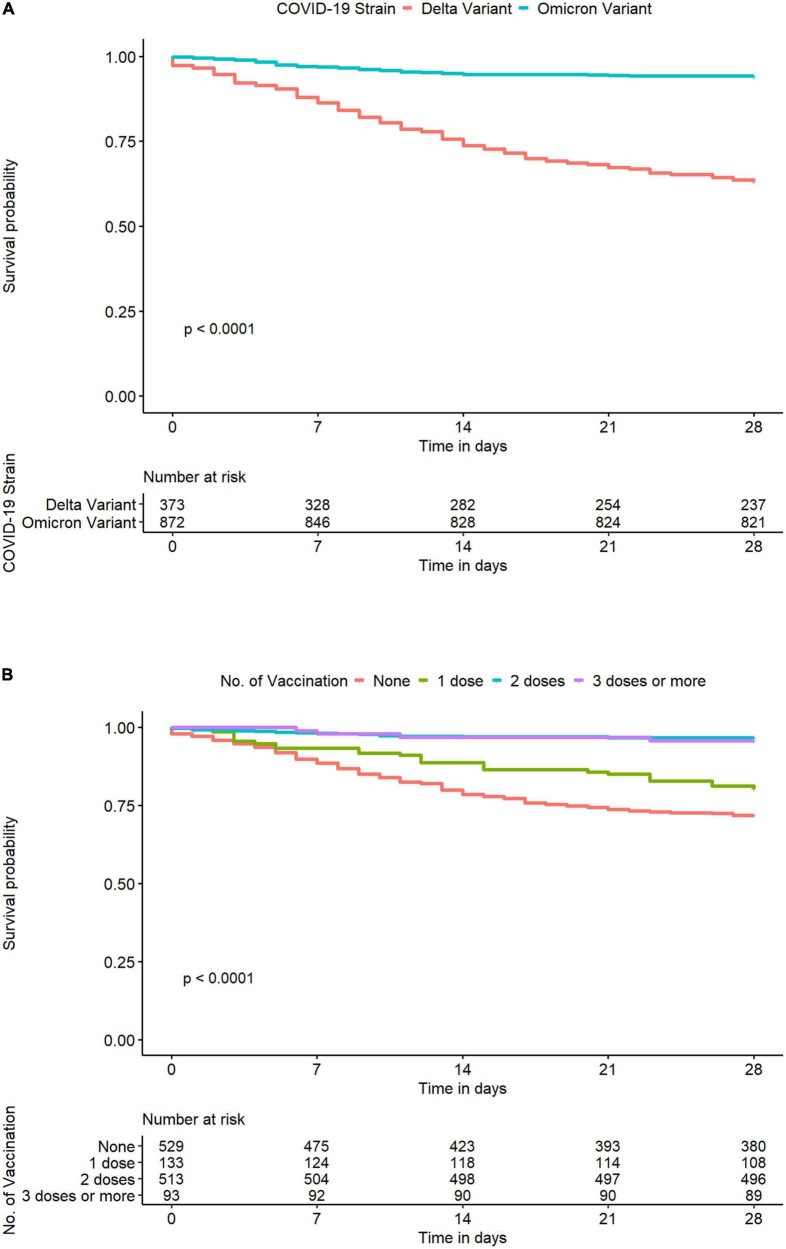
Kaplan–Meier curve for COVID-19-related death within 28 days after COVID-19 diagnosis in Thai PD patients from the InCov19-PD registry, stratified by identified SARS-CoV-2 variants **(A)** and the number of COVID-19 vaccines administered at baseline **(B)** coronavirus disease 2019, COVID-19; SARS-CoV-2, severe acute syndrome coronavirus 2.

On day 28 after COVID-19 diagnosis, 196 (13%) study participants died (non-survivor group), while 1,291 (87%) survived (survivor group). Patients in the non-survivor group were significantly older [mean age (SD): 61.5 (12.6) years vs. 54.8 (14.4) years, *p* < 0.001], had a higher prevalence of diabetes mellitus (69 vs. 52%, *p* < 0.001), and were more functionally dependent (17 vs. 4%, *p* < 0.001) at baseline (Table 1). Those infected with the Delta variant were more likely to die within 28 days after COVID-19 diagnosis than those infected with the Omicron variant (*p* < 0.0001) (Figure 2A). The associations between the number of COVID-19 vaccines administered at baseline and the COVID-19-related death on day 28 after COVID-19 diagnosis are illustrated in Figure 2B. PD patients who were unvaccinated at the time of COVID-19 diagnosis had the highest likelihood of dying within 28 days after COVID-19 diagnosis, followed by those who received one or more doses of COVID-19 vaccine in descending order (*p* < 0.0001) (Figure 2B).

### Predictors associated with death within 28 days of a coronavirus disease 2019 diagnosis

Predictors associated with death within 28 days of a COVID-19 diagnosis are presented in Table 2. Using a time-to-event analysis with a multivariable Cox proportional hazards model, a higher risk of dying within 28 days of a COVID-19 diagnosis among PD patients was independently associated with functional impairment during COVID-19 [adjusted hazard ratio (HR) 2.46, 95% confidence interval (CI) 1.59–3.81], the need for respiratory support (oxygen support: HR 7.13, 95% CI 3.74–13.57) and high flow nasal cannula or intubation (HR 4.21, 95% CI 13.04–5.83), and SARS-CoV-2 infection with the Delta variant (HR 2.23, 95% CI 1.55–3.21) (*p* < 0.05 for all). In contrast, the number of COVID-19 vaccines administered (HR 0.69, 95% CI 0.55–0.87; *p* = 0.001) and corticosteroid treatment during COVID-19 (HR 0.72, 95% CI 0.54–0.97; *p* = 0.03) were associated with a decreased risk of death within 28 days after a COVID-19 diagnosis. Each additional dose of the COVID-19 vaccine administered would reduce the chance of dying within 28 days after COVID-19 diagnosis by approximately 30%. Furthermore, both two-dose homologous and heterologous primary series of COVID-19 vaccination were significantly associated with a lower chance of dying within 28 days after COVID-19 diagnosis in an age-adjusted model (*p* < 0.05 for all). Patients who required respiratory support, whether non-invasive or invasive, were four to seven times more likely to die within 28 days compared to those who did not need oxygen supplementation.

**TABLE 2:** Univariable and multivariable Cox regression models of baseline and during COVID-19 variables associated with death within 28 days after COVID-19 diagnosis.

	Unadjusted model			Age-adjusted model			Multivariable model		
			
	Hazard ratio	95% CI	*p*-value	Hazard ratio	95% CI	*p*-value	Hazard ratio	95% CI	*p*-value
**Baseline**									
Age per 1 year increment	1.04	1.02–1.05	< 0.001	1.04	1.02–1.05	<0.001			
Diabetes	1.90	1.40–2.57	< 0.001	1.59	1.16–2.18	0.004			
Urine output per 100 ml/day increment	0.99	0.95–1.02	0.51	0.98	0.95–1.02	0.38			
PD vintage per 1 year increment	0.97	0.91–1.03	0.34	0.98	0.92–1.05	0.60			
**Assistance with PD bag exchange**									
Partially	2.10	1.56–2.83	< 0.001	1.59	1.13–2.22	0.007			
Completely	2.64	1.56–4.46	< 0.001	2.04	1.17–3.57	0.01			
**Functional status**									
Partially dependent	5.44	3.48–8.50	< 0.001	4.89	3.11–7.71	< 0.001			
Totally dependent	2.39	1.30–4.39	0.005	1.81	0.97–3.37	0.06			
Hemoglobin per 1 g/dL decrement	1.03	0.96–1.11	0.38	1.09	1.01–1.18	0.03			
Serum albumin per 1 g/dL decrement	1.73	1.40–2.14	< 0.001	1.60	1.29–2.00	< 0.001			
Number of COVID-19 vaccines per 1 dose increment	0.38	0.31–0.46	< 0.001	0.38	0.32–0.47	< 0.001	0.69	0.55–0.87	0.001
**Number and regimen of COVID-19 vaccines**									
Single dose	0.65	0.43–0.98	0.04	0.64	0.42–0.97	< 0.001			
Two doses	0.10	0.06–0.17	< 0.001	0.10	0.06–0.17	< 0.001			
Two doses of the inactivated SARS-CoV-2 vaccine	0.17	0.05–0.53	0.002	0.20	0.06–0.63	0.006			
Two doses of the viral vector-based vaccine	0.08	0.03–0.21	< 0.001	0.08	0.03–0.20	< 0.001			
Two doses of the mRNA-based vaccine	0.05	0.01–0.35	0.003	0.06	0.01–0.40	0.004			
Any heterologous vaccination combination	0.12	0.06–0.23	< 0.001	0.12	0.06–0.24	< 0.001			
Three or more doses of any vaccination combination	0.13	0.05–0.36	< 0.001	0.14	0.05–0.38	< 0.001			
At least one dose of the mRNA-based vaccine	0.25	0.10–0.60	0.002	0.27	0.11–0.65	0.004			
**During COVID-19**									
Assistance with PD bag exchange									
Caregiver	3.20	2.02–5.06	< 0.001	3.05	1.88–4.97	< 0.001			
Nurse	13.85	9.16–20.93	< 0.001	13.25	8.48–20.70	< 0.001			
**Functional status**									
Partially dependent	5.04	3.57–7.12	< 0.001	4.78	3.30–6.94	< 0.001	1.89	1.31–2.73	< 0.001
Totally dependent	12.15	8.08–18.25	< 0.001	11.31	7.29–17.55	< 0.001	2.46	1.59–3.81	< 0.001
COVID-19 Delta strain	7.41	5.37–10.22	< 0.001	7.45	5.34–10.41	< 0.001	2.23	1.55–3.21	< 0.001
**Treatment**									
Oxygen support	24.43	13.92–42.89	< 0.001	24.22	13.48–43.52	< 0.001	7.13	3.74–13.57	< 0.001
High flow nasal cannula or intubation	17.24	12.90–23.03	< 0.001	15.96	11.85–21.51	< 0.001	4.21	3.04–5.83	< 0.001
Corticosteroid	2.54	1.92–3.36	< 0.001	2.40	1.80–3.21	< 0.001	0.72	0.54–0.97	0.03
Hospitalization	26.08	10.73–63.38	< 0.001	22.50	9.25–54.74	<0.001			

SARS-CoV-2, severe acute syndrome coronavirus 2; BMI, body mass index; CI, confidence intervals; COVID-19; mRNA, messenger ribonucleic acid; PD, peritoneal dialysis.

### Impacts of coronavirus disease 2019 on hospital course, patient’s functional status, and patterns of peritoneal dialysis bag exchange

Of 1,487 PD patients diagnosed with COVID-19, a total of 921 (62%) patients were hospitalized. Approximately 64% of these patients needed oxygen or other invasive respiratory support (Table 1). More patients infected with the Delta variant required hospitalization and respiratory support than those infected with the Omicron strain [(91 vs. 49%, *p* < 0.001) and (70 vs. 28%, *p* < 0.001), respectively]. At baseline, most (94%) PD patients in this cohort received continuous ambulatory PD (CAPD). During COVID-19, 46 (6%) patients switched from CAPD to automated PD (APD), while all APD patients continued their initial dialysis modality.

The functional status of PD patients with COVID-19 was altered. The number of PD patients who were classified as independent in their activities of daily living (ADLs) declined from 94% at baseline to 63% after COVID-19 diagnosis (*p* < 0.001). Furthermore, the impaired functional status during COVID-19, but not at baseline, was associated with the risk of dying within 28 days from a COVID-19 diagnosis (*p* < 0.001) in the multivariable model. Similarly, COVID-19 impacted the bag exchange patterns of PD patients. At baseline, more than half (59%) of PD patients could independently perform PD bag exchange without assistance. During COVID-19, 10% of patients who were previously independent of PD bag exchange required additional assistance from caregivers or nurses. In the multivariate model, however, the need for PD bag exchange support during COVID-19 illness was not associated with the risk of dying within 28 days from a COVID-19 diagnosis.

## Discussion

In this large, prospective, national observational study, we explored the impact of COVID-19 on the 28-day case fatality rate, predictors associated with death within 28 days of a COVID-19 diagnosis, and changes in functional status and PD bag exchange pattern among PD patients diagnosed with COVID-19 between August 2021 and July 2022. This study revealed a high 28-day COVID-19-related case fatality rate of 13% in PD patients, particularly among unvaccinated patients, those whose functional status declined during COVID-19 illness, those infected with the Delta variant, and those who needed respiratory support. The number of COVID-19 vaccines administered to PD patients at baseline was significantly associated with a lower chance of dying within 28 days of the COVID-19 diagnosis. In addition, independent of baseline functional level, the functional status of PD patients diagnosed with COVID-19 deteriorated significantly during illness, with one-tenth requiring the assistance of caregivers or nurses for PD bag exchange.

The early all-cause fatality rate for ESKD patients requiring KRT with COVID-19 varies widely between 18 and 32% (9, 15–20). Since HD patients and KT recipients were the primary populations investigated in the initial trials, the reported fatality rates represented fatality rates in those populations but did not accurately reflect the fatality rate of the PD population. The COVID-19-related fatality rate among PD patients is poorly understood. Most studies included only a limited number of PD patients, ranging from two to thirty in each trial (2, 13, 20–23). We demonstrated that the early COVID-19-related fatality rate for PD patients was at least comparable to those of PD patients from the other studies (2, 13, 20–23). In a large multicenter European registry consisting of 768 patients on maintenance dialysis and only 4% of study participants on chronic PD therapy, Hillbrands et al. observed a fatality rate of 30% in the PD group at 28 days after COVID-19 diagnosis, compared to a fatality rate of 25% for all dialysis patients (2). Other smaller series, each containing fewer than 10 PD patients, demonstrated a slightly higher all-cause fatality rate among PD patients with COVID-19, ranging from 20 to 31% (20–23). In comparison to the pooled estimated fatality rates in the HD or KT populations from systematic reviews and other large dialysis cohorts (8, 9, 18, 24), the early fatality rate of the PD patients in the present study was lower. The reduced COVID-19-related fatality rate among the PD patients studied here may be attributable to a combination of favorable patient characteristics (8, 9, 20). Older age is consistently recognized as a risk factor for COVID-19-related death in both the general population and ESKD patients (9, 25). However, the PD patients in this cohort were relatively young, had a good baseline functional level, and had a short dialysis vintage. Half of the PD patients in this cohort had received at least two doses of COVID-19 vaccines. Furthermore, the majority of PD patients (70%) were infected with the Omicron variants, which are associated with a lower virulence and disease severity compared to the Delta variant (26, 27). However, direct comparisons of fatality rates of PD patients between studies or between PD patients and HD or KT populations are challenging due to the heterogeneity of the research population, the timing for mortality evaluation from the COVID-19 diagnosis, vaccination status, the SARS-CoV-2 variants that circulated locally, and bed capacities. Moreover, there are differing definitions of fatality outcomes; hence, the fatality rates presented in certain studies may reflect the overall fatality rate rather than the fatality rate attributable exclusively to SARS-CoV-2 infection. The hospitalization rates for confirmed COVID-19 ESKD patients also vary widely across studies between 33% and 88% (14). The disproportionately high hospitalization rate compared to the fatality rate observed in the present study may be partially influenced by Thailand’s national health policy mandating hospitalization for all ESKD patients diagnosed with COVID-19 between January 2020 and March 2022.

Predictors associated with death within 28 days from the COVID-19 diagnosis onset in this study are consistent with those previously described in HD or KT patients. Immunization against SARS-CoV-2 can prevent SARS-CoV-2 infection and reduce the fatality rate in chronic HD patients, with the inactivated SARS-CoV-2 vaccine appearing to elicit weaker immunogenicity and lower clinical protection compared to the mRNA-based vaccine (28–31). Our finding highlighted that a similar association between COVID-19 vaccinations and COVID-19 mortality reduction existed in the PD population, regardless of the vaccine regimen. The protective effect of the COVID-19 vaccination was detected after the first dosage, and the higher the number of doses administered, the lower chance of dying was observed. Functional impairment during COVID-19 and the need for respiratory support, which may reflect comorbidities and the severity of COVID-19, were associated with an increased risk of dying within 28 days after COVID-19 diagnosis, supporting earlier findings in the HD and KT populations (8, 9, 29). In univariate analysis, corticosteroid treatment during COVID-19 was associated with an increased risk of death. However, after adjusting for the number of COVID-19 vaccinations administered, the need for respiratory support, and the presence of comorbidities, corticosteroid treatment was a protective factor for a lower risk of death within 28 days after COVID-19 diagnosis among PD patients. Therefore, we propose that vaccination against COVID-19 should be prioritized in the PD population. Furthermore, corticosteroid treatment should be offered to PD patients with COVID-19 when indicated. Patients who are frail and need oxygen supplementation may benefit from hospitalization and additional monitoring due to their increased risk of adverse outcomes.

Peritoneal dialysis patients diagnosed with COVID-19 experience different challenges compared to HD patients. While HD patients can continue dialysis treatment in hospitals, certain PD patients may be required to undertake dialysis on their own. Fatigue and myalgia are prevalent among HD patients diagnosed with COVID-19 (14, 32). Those symptoms may hinder PD patients’ ability to perform PD bag exchange. Although most PD patients in this cohort could independently perform their PD treatment during COVID-19, more patients (10%) who could previously manage PD bag exchange themselves required support from caregivers or nurses during COVID-19. Therefore, physicians and PD nurses should reevaluate the patient’s ability to self-perform PD treatment if the patient is hospitalized, or discuss this issue with the patient and caregiver if the patient is treated as an outpatient. Furthermore, we showed that functional decline during COVID-19 was prevalent among PD patients and associated with a higher risk of dying within 28 days after COVID-19 diagnosis. The duration of functional impairment following recovery from COVID-19 and the extent to which this may affect PD-related long-term outcomes, such as mortality or peritonitis rate, deserves more investigation.

This is one of the largest nationwide studies investigating the impact of COVID-19 on the fatality rate and clinical outcomes of PD patients. We also addressed the impact of COVID-19 on changes in functional status and the pattern of PD bag exchange, which may have implications for the management and home care of PD patients. Moreover, the protective benefits of COVID-19 vaccination were demonstrated across all major vaccine platforms available to ESKD patients, including the inactivated SARS-CoV-2, the viral-vectored, and the mRNA-based COVID-19 vaccine.

Limitations of our study included the assessment of a COVID-19-related fatality rate over the short term and the absence of an interval between COVID-19 vaccination and COVID-19 diagnosis. Although several studies have indicated that the median duration of COVID-19-related death in ESKD patients occurred during the first month (3, 9), the long-term COVID-19-related fatality rates may differ from the rate determined in this study. In addition, the observed COVID-19-related fatality rate was derived from Thai PD patients infected with SARS-CoV-2 between August 2021 and July 2022, when the Delta and Omicron variants predominated. Therefore, it is possible that the COVID-19-related fatality rates in other countries, where various SARS-CoV-2 variants are a concern, may differ from the COVID-related fatality rate identified here. Since we did not screen all PD patients at each participating site for SARS-CoV-2 infection, we were unable to determine whether the COVID-19 vaccination could reduce the incidence of asymptomatic or symptomatic SARS-CoV-2 infection in this population. Additionally, we were unable to explore the relationship between COVID-19 vaccination intervals and clinical outcomes in this cohort because of limited data on the timing between COVID-19 vaccination and COVID-19 diagnosis. Those may also have confounded the relationship between the numbers of COVID-19 vaccination and fatality outcomes. Although the immunogenicity induced by COVID-19 vaccinations generally wanes over time (33, 34), this does not imply that clinical protection against severity or mortality from COVID-19 has diminished (29). In this study, we described changes in PD bag exchange patterns and the functional status of PD patients during COVID-19. However, we did not evaluate PD prescription or patients’ functional status after COVID-19 recovery. More attention should be given to the impact of functional impairment during COVID-19 and long-term PD-related clinical outcomes.

This study has demonstrated that the COVID-19-related fatality rate at 28 days in PD patients was substantial, but at least comparable to that of HD and KT patients. The predictors of COVID-19-related death in PD patients did not differ from those in HD patients. Moreover, functional impairment during COVID-19 was prevalent among PD patients, and certain patients may require additional help for PD bag exchange during their illness. All measures, especially COVID-19 vaccination, should be prioritized to prevent SARS-CoV-2 infection or its related complications in this vulnerable group. Healthcare professionals should address concerns that may arise as a result of functional deterioration in PD patients diagnosed with COVID-19, which may affect routine PD therapy. The impacts of COVID-19 on long-term PD-related outcomes, including mortality, quality of life, and peritonitis rate warrant additional studies.

## Data availability statement

The datasets presented in this article are not readily available because of privacy and ethical restrictions. Requests to access the datasets should be directed to TK, golfnephro@hotmail.com.

## Ethics statement

The studies involving human participants were reviewed and approved by the Institutional Review Board of the Faculty of Medicine, Chulalongkorn University, Bangkok, Thailand (IRB No. 0298/65). The patients/participants provided their written informed consent to participate in this study.

## Author contributions

PC, PD, SB, SK, SS, and TK conceptualized and designed the study. PC, SB, and TK collected the data and drafted the manuscript. PC, SB, TH, TN, and TK analyzed the data. All authors reviewed and approved a final version of the manuscript.
